# Effects of previous infection, vaccination, and hybrid immunity against symptomatic Alpha, Beta, and Delta SARS-CoV-2 infections: an observational study

**DOI:** 10.1016/j.ebiom.2023.104734

**Published:** 2023-07-27

**Authors:** Heba N. Altarawneh, Hiam Chemaitelly, Houssein H. Ayoub, Patrick Tang, Mohammad R. Hasan, Hadi M. Yassine, Hebah A. Al-Khatib, Asmaa A. Al Thani, Peter Coyle, Zaina Al-Kanaani, Einas Al-Kuwari, Andrew Jeremijenko, Anvar Hassan Kaleeckal, Ali Nizar Latif, Riyazuddin Mohammad Shaik, Hanan F. Abdul-Rahim, Gheyath K. Nasrallah, Mohamed Ghaith Al-Kuwari, Adeel A. Butt, Hamad Eid Al-Romaihi, Mohamed H. Al-Thani, Abdullatif Al-Khal, Roberto Bertollini, Laith J. Abu-Raddad

**Affiliations:** a Infectious Disease Epidemiology Group, Weill Cornell Medicine-Qatar, Cornell University, Doha, Qatar; b World Health Organization Collaborating Centre for Disease Epidemiology Analytics on HIV/AIDS, Sexually Transmitted Infections, and Viral Hepatitis, Weill Cornell Medicine–Qatar, Cornell University, Qatar Foundation – Education City, Doha, Qatar; c Department of Population Health Sciences, Weill Cornell Medicine, Cornell University, New York, NY, USA; d Mathematics Program, Department of Mathematics, Statistics, and Physics, College of Arts and Sciences, Qatar University, Doha, Qatar; e Department of Pathology, Sidra Medicine, Doha, Qatar; f Biomedical Research Center, QU Health, Qatar University, Doha, Qatar; g Department of Biomedical Science, College of Health Sciences, QU Health, Qatar University, Doha, Qatar; h Hamad Medical Corporation, Doha, Qatar; i Wellcome-Wolfson Institute for Experimental Medicine, Queens University, Belfast, United Kingdom; j Department of Public Health, College of Health Sciences, QU Health, Qatar University, Doha, Qatar; k Primary Health Care Corporation, Doha, Qatar; l Department of Medicine, Weill Cornell Medicine, Cornell University, New York, NY, USA; m Ministry of Public Health, Doha, Qatar; n College of Health and Life Sciences, Hamad Bin Khalifa University, Doha, Qatar

**Keywords:** COVID-19, Booster, Reinfection, Variant, Case-control, Test-negative

## Abstract

**Background:**

Protection against SARS-CoV-2 symptomatic infection and severe COVID-19 of previous infection, mRNA two-dose vaccination, mRNA three-dose vaccination, and hybrid immunity of previous infection and vaccination were investigated in Qatar for the Alpha, Beta, and Delta variants.

**Methods:**

Six national, matched, test-negative, case-control studies were conducted between January 18 and December 18, 2021 on a sample of 239,120 PCR-positive tests and 6,103,365 PCR-negative tests.

**Findings:**

Effectiveness of previous infection against Alpha, Beta, and Delta reinfection was 89.5% (95% CI: 85.5–92.3%), 87.9% (95% CI: 85.4–89.9%), and 90.0% (95% CI: 86.7–92.5%), respectively. Effectiveness of two-dose BNT162b2 vaccination against Alpha, Beta, and Delta infection was 90.5% (95% CI, 83.9–94.4%), 80.5% (95% CI: 79.0–82.0%), and 58.1% (95% CI: 54.6–61.3%), respectively. Effectiveness of three-dose BNT162b2 vaccination against Delta infection was 91.7% (95% CI: 87.1–94.7%). Effectiveness of hybrid immunity of previous infection and two-dose BNT162b2 vaccination was 97.4% (95% CI: 95.4–98.5%) against Beta infection and 94.5% (95% CI: 92.8–95.8%) against Delta infection. Effectiveness of previous infection and three-dose BNT162b2 vaccination was 98.1% (95% CI: 85.7–99.7%) against Delta infection. All five forms of immunity had >90% protection against severe, critical, or fatal COVID-19 regardless of variant. Similar effectiveness estimates were observed for mRNA-1273. A mathematical model accurately predicted hybrid immunity protection by assuming that the individual effects of previous infection and vaccination acted independently.

**Interpretation:**

Hybrid immunity, offering the strongest protection, was mathematically predicted by assuming that the immunities obtained from previous infection and vaccination act independently, without synergy or redundancy.

**Funding:**

The Biomedical Research Program and the Biostatistics, Epidemiology, and the Biomathematics Research Core, both at 10.13039/100019460Weill Cornell Medicine-Qatar, Ministry of Public Health, 10.13039/100007833Hamad Medical Corporation, 10.13039/100019475Sidra Medicine, Qatar Genome Programme, Qatar University Biomedical Research Center, and 10.13039/501100004252Qatar University Internal Grant ID QUCG-CAS-23/24-114.

**Research in context**

**Evidence before this study**

Before the emergence of the Omicron variant, both SARS-CoV-2 infection and COVID-19 vaccination were effective in protecting against new infections, although vaccine immunity waned over time. However, the combined immunological effect of previous SARS-CoV-2 infection and COVID-19 vaccination, known as hybrid immunity, in relation to infection with different pre-Omicron variants of the virus, remains inadequately understood. While some studies have reported single measures of hybrid immunity against certain pre-Omicron variants, the relationship between hybrid immunity and each of previous infection and vaccination has yet to be established. A search of PubMed and Google Scholar search engines up to April 15, 2023 using the keywords “vaccination”, “infection”, “reinfection”, “immunity”, “protection”, “SARS-CoV-2”, and “COVID-19” did not yield any studies that provided a detailed investigation of hybrid immunity resulting from previous infection and vaccination against the Alpha, Beta, and Delta variants of the pre-Omicron era, nor did they establish whether hybrid immunity can be mathematically predicted based on the effects of previous infection and vaccination.

**Added value of this study**

This study investigated the interplay of immunity from natural infection and vaccination through an analysis of the national federated databases for SARS-CoV-2 infection and COVID-19 vaccination in Qatar during Alpha, Beta, and Delta incidence, when vaccination was being scaled up. A matched, test-negative, study design was used to investigate protection of previous infection, of mRNA vaccination after the second dose and after the third/booster dose, and of hybrid immunity combining previous infection and vaccination against both infection and severe COVID-19. All forms of natural and vaccine immunity provided strong protection against Alpha, Beta, and Delta infections, and very strong protection against severe COVID-19. Yet, hybrid immunity of natural infection and vaccination provided consistently higher protection against infection and severe COVID-19 than each of natural infection or vaccination alone, regardless of variant or mRNA vaccine type. The level of hybrid immunity protection was strikingly very similar to that predicted using a mathematical model assuming that the immunological effects of previous infection and vaccination act independently of each other. The model predicted, with precision, the level of hybrid immunity protection not only against Alpha, Beta, and Delta, but also against BA.1 and BA.2 Omicron subvariants.

**Implications of all the available evidence**

Hybrid immunity offers the most robust protection and its protection can be mathematically predicted in the first few months following infection and vaccination. This prediction is based on the assumption that the acquired immunities from infection and vaccination act independently, without any synergy or redundancy. Consistently observed across different variants, both before and after the emergence of Omicron, and regardless of vaccine type and dose number, this finding suggests the existence of a generic immunological pattern that applies to SARS-CoV-2-generated immunity. This pattern may hold potential implications for optimizing protection against infection and severe COVID-19 through the development of diverse vaccine and booster formulations. These findings underscore the importance of vaccination, even for individuals with a prior SARS-CoV-2 infection.

## Introduction

Prior to introduction of severe acute respiratory syndrome coronavirus 2 (SARS-CoV-2) Omicron (B.1.1.529) variant in December of 2021,^1^ Qatar experienced three waves of infection dominated sequentially by the original virus,^2^ Alpha (B.1.1.7) variant,^3^ and Beta (B.1.351) variant (Supplementarty Fig. S1 of Supplementary Appendix).^4^ These waves were followed by a prolonged low-incidence phase dominated by the Delta (B.1.617.2) variant (Supplementarty Fig. S1).^5^^,^^6^ The Alpha and Beta waves and the early phase of Delta incidence coincided with the rapid scale-up of coronavirus disease 2019 (COVID-19) vaccination using the BNT162b2 (Pfizer-BioNTech)^7^ and mRNA-1273 (Moderna)^8^ vaccines.^9^

These dynamics provide an opportunity to comprehensively investigate the interplay of the effects of previous infection and vaccination against symptomatic Alpha, Beta, and Delta infections. We estimated protection of previous infection, of mRNA vaccination after the second dose and after the third/booster dose, and of hybrid immunity of previous infection and vaccination against infection with these variants as well as against any severe (acute-care hospitalization),^10^ critical (intensive-care-unit hospitalization),^10^ or fatal^11^ COVID-19. We also investigated whether hybrid immunity effect can be mathematically predicted from the individual effects of each of previous infection and vaccination.

## Methods

### Study population and data sources

This study was conducted on the population of Qatar between January 18, 2021, onset of the Alpha wave,^3^ through December 18, 2021, right before onset of the Omicron wave on December 19, 2021.^1^ The study analyzed the national, federated databases for COVID-19 laboratory testing, vaccination, hospitalization, and death, retrieved from the integrated, nationwide, digital-health information platform (Supplementarty Text S1). Databases include all SARS-CoV-2-related data with no missing information since onset of the pandemic, including all polymerase chain reaction (PCR) tests regardless of location or facility (Supplementarty Text S2). SARS-CoV-2 testing during the study was widely available and performed extensively, mostly for non-clinical reasons.^5^^,^^12^ Most infections were diagnosed not because of symptoms, but because of routine testing.^5^^,^^12^ Demographic information, such as sex and age, were extracted as registered in the national health registry. Sex was taken into account in the design of the study and controls and cases were matched by sex. Further descriptions of Qatar’s population and of the national databases have been reported previously.^5^^,^^12^, ^13^, ^14^, ^15^

### Study design

This study estimated effectiveness of previous pre-Omicron infection, vaccination with BNT162b2 or mRNA-1273, and hybrid immunity against symptomatic infection with Alpha, Beta, or Delta using a test-negative, case-control study design.^1^^,^^16^, ^17^, ^18^ The study design followed that developed earlier to investigate effects of previous infection, vaccination, and hybrid immunity against symptomatic Omicron infections.^12^ This design estimates effectiveness by comparing odds of previous infection and/or vaccination among PCR-positive tests (cases) versus PCR-negative tests (controls).^1^^,^^16^, ^17^, ^18^ To optimize the precision of estimates, the study included all eligible cases and controls throughout the study duration, obviating the need for sample size calculation.

Every PCR test in Qatar is classified on the basis of symptoms and the reason for testing (clinical symptoms, contact tracing, surveys or random testing campaigns, individual requests, routine healthcare testing, pre-travel, port of entry, or other). This categorization enabled us to distinguish tests conducted due to symptoms. The purpose of this study, for pre-Omicron immunity, was to complement our earlier study that examined Omicron immunity^12^ and to compare the interplay of the immunological effects of previous infection and vaccination. The earlier study specifically focused on symptomatic infection,^12^ and therefore, to maintain consistency and allow comparison of the immunological patterns, per-Omicron and post-Omicron, this study was conducted also against symptomatic infection.

In estimating effectiveness against symptomatic infection, we exactly matched controls to cases two-to-one by sex, 10-year age group, nationality, number of coexisting conditions (0, 1–2, or ≥3), and calendar week of PCR test. Exact matching refers here to the pairing of controls and cases based on identical values for the matching factors. The matched pairs had precisely the same characteristics. For estimating effectiveness against any severe,^10^ critical,^10^ or fatal^11^ COVID-19, a five-to-one matching ratio was used to enhance statistical precision.

The selection of matching factors in our study was based on previous evidence from Qatar,^19^, ^20^, ^21^, ^22^ which identified factors associated with both the exposure and/or disease.^23^ Through matching, we aimed to achieve balance in these observed confounders.^19^, ^20^, ^21^, ^22^ Furthermore, we implemented matching by calendar week of testing to minimize potential bias arising from variations in the epidemic phase and vaccination rollout during the study.^16^^,^^24^ The choice of matching factors was also informed by the results of prior studies conducted on Qatar's population.^5^^,^^9^^,^^25^, ^26^, ^27^

Only the first PCR-positive test occurring during a variant-dominated period was included for cases, while all PCR-negative tests were included for controls. Controls consisted of PCR-negative tests for individuals with no record of a PCR-positive test during that period. Only PCR tests conducted because of clinical suspicion due to presence of symptoms compatible with a respiratory tract infection were analyzed.

SARS-CoV-2 reinfection is conventionally defined as a documented infection ≥90 days after a previous infection, to avoid misclassification of prolonged PCR positivity as reinfection,^28^ if a shorter time interval is used.^1^^,^^29^^,^^30^ Previous infection was thus defined as a PCR-positive test ≥90 days before this study’s PCR test. Cases or controls with PCR-positive tests <90 days before the study’s PCR test were excluded.

Tests on individuals who received vaccines other than BNT162b2 or mRNA-1273, or who received mixed vaccines, were excluded. Tests occurring within 14 days of a second vaccine dose or 7 days of a third (booster) dose were excluded. These inclusion and exclusion criteria were implemented to allow for immunity build-up after vaccination,^13^^,^^31^ and to minimize different types of potential bias, as informed by earlier analyses on the same population.^5^^,^^18^^,^^27^ Every control that met the inclusion criteria and that could be matched to a case was included.

The study compared five exposure groups who had one or more immunological events of infection and/or vaccination to those with no previous infection and no vaccination. These groups included individuals with only previous infection, only two-dose (primary-series) vaccination, only three-dose (primary-series plus booster) vaccination, previous infection and two-dose vaccination, and previous infection and three-dose vaccination. These groups were defined based on status of prior immunological events at the time of the PCR test.

Classification of COVID-19 case severity,^10^ criticality,^10^ and fatality^11^ followed World Health Organization guidelines, based on a national protocol applied to hospitalized COVID-19 patients (Supplementarty Text S3).

### Variant ascertainment

The variant status of each infection was determined by the variant that dominated incidence at time of infection diagnosis. Duration of dominance of each variant was per Qatar’s variant genomic surveillance.^32^, ^33^, ^34^ This surveillance consists of viral genome sequencing^32^ and multiplex real-time reverse-transcription PCR (RT-qPCR) variant screening^33^ of weekly collected random positive clinical samples, complemented by deep sequencing of wastewater samples^34^ (Supplementarty Text S2). Accordingly, an Alpha, Beta, or Delta infection was proxied as an infection diagnosed during January 18-March 7, 2021, March 8-May 31, 2021, or May 31-December 18, 2021, respectively.

### Statistical analysis

While all records of PCR testing were examined for selection of cases and controls, only matched samples were analyzed. Cases and controls were described using frequency distributions and measures of central tendency and compared using standardized mean differences (SMDs). An SMD of ≤0.1 indicated adequate matching.^35^ The “stddiff” command in STATA was employed to calculate the SMDs for categorical variables. It accommodated multiple categories of these variables by treating them as factor variables.^36^

Odds ratios, comparing odds of previous infection and/or vaccination among cases versus controls, and associated 95% confidence intervals (CIs) were derived using conditional logistic regression. Other than the matching factors, no additional observable confounders were deemed necessary for inclusion in the conditional logistic regression model. CIs were not adjusted for multiplicity and interactions were not investigated. When conditional logistic regression failed to converge due to zero matched pairs in a specific exposure category, the 95% CI was calculated using McNemar’s test. Since n:1 matching was employed, the number of pairs was considered as ‘n’. This approach provided only an approximate estimate for the 95% CI in these specific situations. We encountered limitations in using other methods in Stata, such as penalized conditional logistic regression, to obtain estimates for the 95% CI.

The reference group for all estimates comprised individuals with no previous infection and no vaccination. Based on the methodology of the test-negative design,^16^^,^^18^ effectiveness measures and associated 95% CIs were calculated as 1-odds ratio of previous infection and/or vaccination among cases versus controls (Supplementarty Text S1). Statistical analyses were conducted in STATA/SE version 17.0 (Stata Corporation, College Station, TX, USA).

Estimated effectiveness of hybrid immunity of previous infection and vaccination was compared to predicted effectiveness of hybrid immunity of previous infection and vaccination as calculated assuming that the effects of each of previous infection and vaccination act independently. If these two immunity effects are independent, effectiveness of hybrid immunity (z) is given by z=1−(1−x)×(1−y), where x is effectivensss of previous infection alone and y is effecteivness of vaccination alone.

This independence-model effectiveness was calculated against Alpha, Beta, and Delta as well as against any Omicron subvariant and against BA.1 and BA.2 Omicron subvariants. The estimates for previous infection and vaccination specific to the Omicron subvariants were obtained from a prior publication by Altarawneh et al.^12^ These estimates were utilized in our study to examine the consistency between the earlier estimations for hybrid immunity against Omicron subvariants^12^ and the predictions made by the independence model.

Considering the mathematical definitions of synergy and redundancy for individual effects,^37^ the comparison between the estimated effectiveness of hybrid immunity and the prediction of the independence model offers insights into the interaction between natural infection immunity and vaccine immunity. If the estimated effectiveness of hybrid immunity is higher than that estimated using the independence model, it implies that the combined effect of natural infection immunity and vaccine immunity is synergistic.^37^ Their combination produces a result that is greater than what would be expected based on the individual effects alone. The synergy between natural infection immunity and vaccine immunity amplifies their individual effects.

Conversely, if the estimated effectiveness of hybrid immunity is lower than that estimated using the independence model, it implies that the combination of natural infection immunity and vaccine immunity exhibits redundancy.^37^ There is duplication or overlap in the effects of these two forms of immunity, resulting in an outcome affected by redundancy.

Agreement between the directly estimated hybrid immunity effectiveness and the independence-model effectiveness was investigated by calculating the interclass correlation coefficient (ICC).^38^ The ICC estimate and its confidence interval were calculated using Stata based on individual-rater type, absolute agreement, and 2-way random effects model.^38^

### Ethical approval and oversight

Hamad Medical Corporation (HMC IRB number: MRC-01-20-1078) and Weill Cornell Medicine–Qatar (WCM-Q IRB number: 20–00017) Institutional Review Boards approved this retrospective study with a waiver of informed consent. The study was reported according to the Strengthening the Reporting of Observational Studies in Epidemiology (STROBE) guidelines (Supplementarty Table S1).

The dataset of this study is a property of the Qatar Ministry of Public Health that was provided to the researchers through a restricted-access agreement that prevents sharing the dataset with a third party or publicly. The data are available under restricted access for preservation of confidentiality of patient data.

### Role of the funding source

The funders of the study had no role in study design, data collection, data analysis, data interpretation, or writing of the report. The corresponding authors had full access to all the data in the study and had final responsibility for the decision to submit for publication.

## Results

### Study population

Between December 23, 2020, date of first vaccination in Qatar,^31^ and December 18, 2021, end of study, 1,286,955 individuals received at least two BNT162b2 doses, of whom 152,316 received a third (booster) dose. The median date was May 2, 2021 for the first dose, May 23, 2021 for the second dose, and November 25, 2021 for the third dose. The median duration between the first and second doses was 21 days (interquartile range (IQR), 21–22 days), and the median duration between the second and third doses was 247 days (IQR, 238–258 days).

Meanwhile, 887,773 individuals received at least two mRNA-1273 doses, of whom 26,598 received a third dose. The median date was May 27, 2021 for the first dose, June 27, 2021 for the second dose, and December 6, 2021 for the third dose. The median duration between the first and second doses was 28 days (IQR, 28–30), and between the second and third doses was 216 days (IQR, 207–225).

Supplementarty Figs. S2 and S3 show the selection process of study populations for each of the BNT162b2 and mRNA-1273 analyses, respectively. Table 1 and Supplementarty Table S2 describe the characteristics of study populations for these analyses, respectively.

**Table 1:** Characteristics of matched cases and controls in samples used to estimate effectiveness against symptomatic Alpha, Beta, or Delta infections in the BNT162b2 analysis.

Characteristics	Effectiveness against symptomatic Alpha infection			Effectiveness against symptomatic Beta infection			Effectiveness against symptomatic Delta infection		
	Cases^a^, (PCR-positive)	Controls^a^, (PCR-negative)	SMD^b^	Cases^a^, (PCR-positive)	Controls^a^, (PCR-negative)	SMD^b^	Cases^a^, (PCR-positive)	Controls^a^, (PCR-negative)	SMD^b^
	N = 7178	N = 13,821		N = 19,019	N = 36,544		N = 7201	N = 13,954	
Median age (IQR) — years	33 (22–41)	33 (22–40)	0.02^c^	32 (20–39)	31 (21–39)	0.02^c^	29 (11–39)	28 (11–39)	0.01^c^
Age group — no. (%)									
<10 years	715 (10.0)	1382 (10.0)	0.02	2491 (13.1)	4791 (13.1)	0.02	1536 (21.3)	3009 (21.6)	0.02
10–19 years	850 (11.8)	1629 (11.8)		2086 (11.0)	4001 (10.9)		1135 (15.8)	2211 (15.8)	
20–29 years	1293 (18.0)	2506 (18.1)		3767 (19.8)	7332 (20.1)		1056 (14.7)	2063 (14.8)	
30–39 years	2330 (32.5)	4556 (33.0)		6031 (31.7)	11,758 (32.2)		1760 (24.4)	3449 (24.7)	
40–49 years	1361 (19.0)	2613 (18.9)		3059 (16.1)	5814 (15.9)		1092 (15.2)	2093 (15.0)	
50–59 years	485 (6.8)	886 (6.4)		1098 (5.8)	2000 (5.5)		398 (5.5)	728 (5.2)	
60–69 years	110 (1.5)	188 (1.4)		342 (1.8)	590 (1.6)		159 (2.2)	283 (2.0)	
70+ years	34 (0.5)	61 (0.4)		145 (0.8)	258 (0.7)		65 (0.9)	118 (0.8)	
Sex									
Male	3981 (55.5)	7697 (55.7)	0.00	10,985 (57.8)	21,135 (57.8)	0.00	3680 (51.1)	7129 (51.1)	0.00
Female	3197 (44.5)	6124 (44.3)		8034 (42.2)	15,409 (42.2)		3521 (48.9)	6825 (48.9)	
Nationality^d^									
Bangladeshi	319 (4.4)	622 (4.5)	0.04	1227 (6.5)	2363 (6.5)	0.04	271 (3.8)	529 (3.8)	0.04
Egyptian	746 (10.4)	1459 (10.6)		1294 (6.8)	2464 (6.7)		753 (10.5)	1476 (10.6)	
Filipino	708 (9.9)	1384 (10.0)		1965 (10.3)	3815 (10.4)		376 (5.2)	729 (5.2)	
Indian	1723 (24.0)	3394 (24.6)		3358 (17.7)	6607 (18.1)		738 (10.2)	1461 (10.5)	
Nepalese	268 (3.7)	524 (3.8)		987 (5.2)	1925 (5.3)		103 (1.4)	202 (1.4)	
Pakistani	323 (4.5)	602 (4.4)		658 (3.5)	1232 (3.4)		175 (2.4)	334 (2.4)	
Qatari	1371 (19.1)	2731 (19.8)		4840 (25.4)	9662 (26.4)		2875 (39.9)	5729 (41.1)	
Sri Lankan	154 (2.1)	291 (2.1)		510 (2.7)	957 (2.6)		52 (0.7)	99 (0.7)	
Sudanese	211 (2.9)	390 (2.8)		605 (3.2)	1128 (3.1)		201 (2.8)	376 (2.7)	
Other nationalities^e^	1355 (18.9)	2424 (17.5)		3575 (18.8)	6391 (17.5)		1657 (23.0)	3019 (21.6)	
Coexisting conditions									
0	5080 (70.8)	9927 (71.8)	0.03	13,925 (73.2)	27,148 (74.3)	0.03	4875 (67.7)	9545 (68.4)	0.02
1	1163 (16.2)	2194 (15.9)		2985 (15.7)	5573 (15.3)		1379 (19.2)	2651 (19.0)	
2	480 (6.7)	869 (6.3)		1026 (5.4)	1833 (5.0)		477 (6.6)	871 (6.2)	
3+	455 (6.3)	831 (6.0)		1083 (5.7)	1990 (5.4)		470 (6.5)	887 (6.4)	
PCR test calendar month^f^									
January	1442 (20.1)	2787 (20.2)	0.00	0 (0.0)	0 (0.0)	0.01	0 (0.0)	0 (0.0)	0.02
February	4397 (61.3)	8454 (61.2)		0 (0.0)	0 (0.0)		0 (0.0)	0 (0.0)	
March	1339 (18.7)	2580 (18.7)		7655 (40.2)	14,844 (40.6)		0 (0.0)	0 (0.0)	
April	0 (0.0)	0 (0.0)		9347 (49.1)	17,818 (48.8)		0 (0.0)	0 (0.0)	
May	0 (0.0)	0 (0.0)		2017 (10.6)	3882 (10.6)		0 (0.0)	0 (0.0)	
June	0 (0.0)	0 (0.0)		0 (0.0)	0 (0.0)		814 (11.3)	1593 (11.4)	
July	0 (0.0)	0 (0.0)		0 (0.0)	0 (0.0)		883 (12.3)	1656 (11.9)	
August	0 (0.0)	0 (0.0)		0 (0.0)	0 (0.0)		1428 (19.8)	2804 (20.1)	
September	0 (0.0)	0 (0.0)		0 (0.0)	0 (0.0)		864 (12.0)	1666 (11.9)	
October	0 (0.0)	0 (0.0)		0 (0.0)	0 (0.0)		622 (8.6)	1261 (9.0)	
November	0 (0.0)	0 (0.0)		0 (0.0)	0 (0.0)		1484 (20.6)	2845 (20.4)	
December	0 (0.0)	0 (0.0)		0 (0.0)	0 (0.0)		1106 (15.4)	2129 (15.3)	

IQR denotes interquartile range, PCR polymerase chain reaction, and SMD standardized mean difference.

a Cases and controls were matched exactly one-to-two by sex, 10-year age group, nationality, number of coexisting conditions, and calendar week of PCR test.

b SMD is the difference in the mean of a covariate between groups divided by the pooled standard deviation. An SMD of ≤0.1 indicates adequate matching.

c Here we reported the median, but the SMD was calculated for the mean difference between groups divided by the pooled standard deviation.

d Nationalities were chosen to represent the most populous groups in Qatar.

e These comprise 50 other nationalities in Qatar among cases and controls in the analysis for effectiveness against symptomatic Alpha infection, 68 other nationalities among cases and controls in the analysis for effectiveness against symptomatic Beta infection, and 40 other nationalities among cases and controls in the analysis for effectiveness against symptomatic Delta infection.

f Cases and controls were matched exactly using calendar week of PCR test, but we opted to report the distribution by calendar month for brevity. Accordingly, some cases and controls who were tested in the same week may appear in different calendar months.

### Effectiveness against symptomatic Alpha infection

#### BNT162b2 analysis

**Fig. 1:**
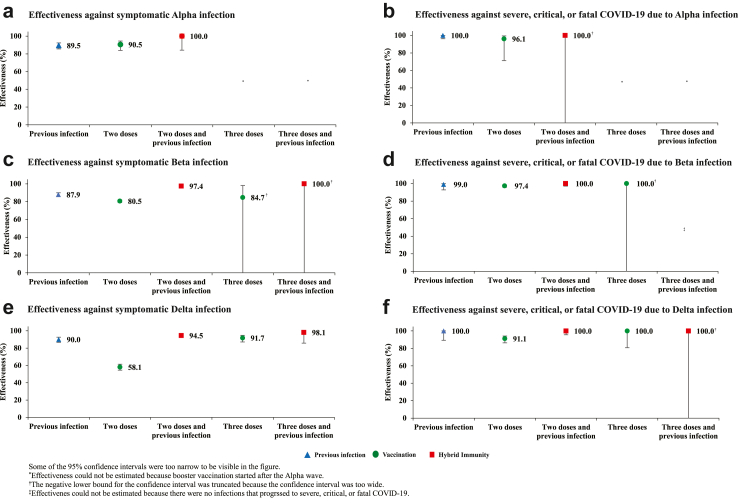
Effectiveness of previous pre-Omicron infection, vaccination with BNT162b2, and hybrid immunity of previous infection and vaccination against symptomatic Alpha, Beta, or Delta infections and against severe, critical, or fatal COVID-19 due to infection with these variants.

Effectiveness of only previous infection against symptomatic Alpha infection was 89.5% (95% CI: 85.5–92.3%) (Fig. 1A and Table 2). The median duration between the previous infection and PCR test was 226.5 days (IQR, 169–257 days). Effectiveness of only two-dose BNT162b2 vaccination was 90.5% (95% CI, 83.9–94.4%). The median duration between the second dose and PCR test was 25 days (IQR, 19–32 days). Effectiveness of hybrid immunity of previous infection and two-dose BNT162b2 vaccination was 100.0% (95% CI: 84.1–100.0%).

**Table 2:** Effectiveness of previous pre-Omicron infection, vaccination with BNT162b2, and hybrid immunity of previous infection and vaccination against symptomatic Alpha, Beta, or Delta infections and against severe, critical, or fatal COVID-19 due to infection with these variants.

Analyses	Cases (PCR-positive)^a^		Controls (PCR-negative)^a^		Effectiveness against symptomatic infection (95% CI)	Cases (Severe, critical, or fatal COVID-19)^b^		Controls (PCR- negative)^b^		Effectiveness against severe, critical, or fatal COVID-19 (95% CI)
	Exposed	Unexposed^c^	Exposed	Unexposed^c^		Exposed	Unexposed^c^	Exposed	Unexposed^c^	
Alpha symptomatic infection^d^										
Previous infection and no vaccination	41	7122	686	12,855	89.5 (85.5–92.3)	0	438	99	1641	100.0 (96.2–100.0)^e^
Two doses and no previous infection	15	7122	255	12,855	90.5 (83.9–94.4)	1	438	74	1641	96.1 (71.2–99.5)
Two doses and previous infection	0	7122	25	12,855	100.0 (84.1–100.0)^e^	0	438	3	1641	100.0 (−58.7 to 100.0)^e^
Three doses and no previous infection	0	7122	0	12,855	–	0	438	0	1641	–
Three doses and previous infection	0	7122	0	12,855	–	0	438	0	1641	–
Beta symptomatic infection^d^										
Previous infection and no vaccination	132	17,738	1631	28,012	87.9 (85.4–89.9)	1	1420	291	4028	99.0 (92.8–99.9)
Two doses and no previous infection	1135	17,738	6376	28,012	80.5 (79.0–82.0)	25	1420	1443	4028	97.4 (95.8–98.4)
Two doses and previous infection	13	17,738	516	28,012	97.4 (95.4–98.5)	0	1420	125	4028	100.0 (97.0–100.0)^e^
Three doses and no previous infection	1	17,738	7	28,012	84.7 (−22.7 to 98.2)	0	1420	1	4028	100.0 (−97.4 to 100.0)^e^
Three doses and previous infection	0	17,738	2	28,012	100.0 (−81.2 to 100.0)^e^	0	1420	0	4028	–
Delta symptomatic infection^d^										
Previous infection and no vaccination	52	4207	654	5844	90.0 (86.7–92.5)	0	189	36	244	100.0 (89.2–100.0)^e^
Two doses and no previous infection	2856	4207	6236	5844	58.1 (54.6–61.3)	59	189	544	244	91.1 (86.3–94.2)
Two doses and previous infection	59	4207	965	5844	94.5 (92.8–95.8)	0	189	85	244	100.0 (95.6–100.0)^e^
Three doses and no previous infection	26	4207	219	5844	91.7 (87.1–94.7)	0	189	21	244	100.0 (80.8–100.0)^e^
Three doses and previous infection	1	4207	36	5844	98.1 (85.7–99.7)	0	189	3	244	100.0 (−58.7 to 100.0)^e^

CI denotes confidence interval, COVID-19 coronavirus disease 2019, and PCR polymerase chain reaction.

a Cases and controls were exactly matched one-to-two by sex, 10-year age group, nationality, number of coexisting conditions, and calendar week of PCR test.

b Cases and controls were exactly matched one-to-five by sex, 10-year age group, nationality, number of coexisting conditions, and calendar week of PCR test.

c Unexposed was defined as no previous infection and no vaccination.

d A symptomatic infection was defined as a PCR-positive nasopharyngeal swab that was obtained because of the presence of symptoms consistent with a respiratory tract infection. Effectiveness was estimated with the use of a test-negative, case-control study design. COVID-19 severity, criticality, and fatality were defined according to World Health Organization guidelines.

e The 95% confidence interval was estimated with the use of McNemar’s test because of zero events among exposed cases. Since n:1 matching was employed, the number of pairs was considered as ‘n’. This approach provided only an approximate estimate for the 95% CI in these specific situations.

#### mRNA-1273 analysis

**Fig. 2:**
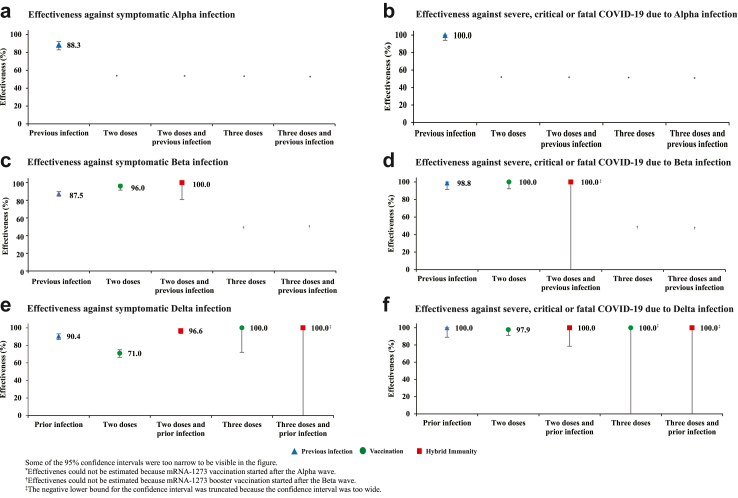
Effectiveness of previous pre-Omicron infection, vaccination with mRNA-1273, and hybrid immunity of previous infection and vaccination against symptomatic Alpha, Beta, or Delta infections and against severe, critical, or fatal COVID-19 due to infection with these variants.

It was not possible to estimate mRNA-1273 effectiveness against Alpha, as number of mRNA-1273 vaccinations was limited. However, it was possible to provide another estimate for effectiveness of only previous infection against symptomatic Alpha infection at 88.3% (95% CI: 82.8–92.1%) (Fig. 2A and Supplementarty Table S3). The median duration between the previous infection and PCR test was 232 days (IQR, 184.5–261 days).

### Effectiveness against symptomatic Beta infection

#### BNT162b2 analysis

Effectiveness of only previous infection against symptomatic Beta infection was 87.9% (95% CI: 85.4–89.9%) (Fig. 1C and Table 2). The median duration between the previous infection and PCR test was 272 days (IQR, 213–307 days).

Effectiveness of only two-dose BNT162b2 vaccination was 80.5% (95% CI: 79.0–82.0%). The median duration between the second dose and PCR test was 35 days (IQR, 23–53 days). Effectiveness of only three-dose BNT162b2 vaccination was 84.7% (95% CI: −22.7–98.2%). The median duration between the third dose and PCR test was 25 days (IQR, 21–34 days).

Effectiveness of hybrid immunity of previous infection and two-dose BNT162b2 vaccination was 97.4% (95% CI: 95.4–98.5%). Effectiveness of previous infection and three-dose BNT162b2 vaccination was 100.0% (95% CI: −81.2–100.0%).

#### mRNA-1273 analysis

Effectiveness of only previous infection against symptomatic Beta infection was 87.5% (95% CI: 84.6–89.9%) (Fig. 2C and Supplementarty Table S3). The median duration between the previous infection and PCR test was 277 days (IQR, 214–308 days). Effectiveness of only two-dose mRNA-1273 vaccination was 96.0% (95% CI, 91.6–98.1%). The median duration between the second dose and PCR test was 21 days (IQR, 17–30 days). Effectiveness of hybrid immunity of previous infection and two-dose mRNA-1273 vaccination was 100.0% (95% CI: 80.8–100.0%).

### Effectiveness against symptomatic Delta infection

#### BNT162b2 analysis

Effectiveness of only previous infection against symptomatic Delta infection was 90.0% (95% CI: 86.7–92.5%) (Fig. 1E and Table 2). The median duration between the previous infection and PCR test was 280 days (IQR, 196–404 days).

Effectiveness of only two-dose BNT162b2 vaccination was 58.1% (95% CI: 54.6–61.3%). The median duration between the second dose and PCR test was 150 days (IQR, 98–204 days). Effectiveness of only three-dose BNT162b2 vaccination was 91.7% (95% CI: 87.1–94.7%). The median duration between the third dose and PCR test was 23.5 days (IQR, 14–43 days).

Effectiveness of hybrid immunity of previous infection and two-dose BNT162b2 vaccination was 94.5% (95% CI: 92.8–95.8%). Effectiveness of previous infection and three-dose BNT162b2 vaccination was 98.1% (95% CI: 85.7–99.7%).

#### mRNA-1273 analysis

Effectiveness of only previous infection against symptomatic Delta infection was 90.4% (95% CI: 86.8–93.0%) (Fig. 2E and Supplementarty Table S3). The median duration between the previous infection and PCR test was 258.5 days (IQR, 186–389 days).

Effectiveness of only two-dose mRNA-1273 vaccination was 71.0% (95% CI: 66.4–74.9%). The median duration between the second dose and PCR test was 111 days (IQR, 59–168 days). Effectiveness of only three-dose mRNA-1273 vaccination was 100.0% (95% CI: 72.1–100.0%). The median duration between the third dose and PCR test was 19 days (IQR, 12–30 days).

Effectiveness of hybrid immunity of previous infection and two-dose mRNA-1273 vaccination was 96.6% (95% CI: 93.5–98.2%). Effectiveness of previous infection and three-dose mRNA-1273 vaccination was 100.0% (95% CI: −8.4-100.0%).

### Effectiveness against severe, critical, or fatal COVID-19

Previous infection, vaccination, and hybrid immunity all showed robust effectiveness (>90%) against severe, critical, or fatal COVID-19 regardless of the underlying variant, but some of the 95% CIs were wide because of small case numbers (Fig. 1, Fig. 2, Table 2, and Supplementarty Table S3).

### Hybrid immunity protection: directly estimated versus independence-model prediction

**Fig. 3:**
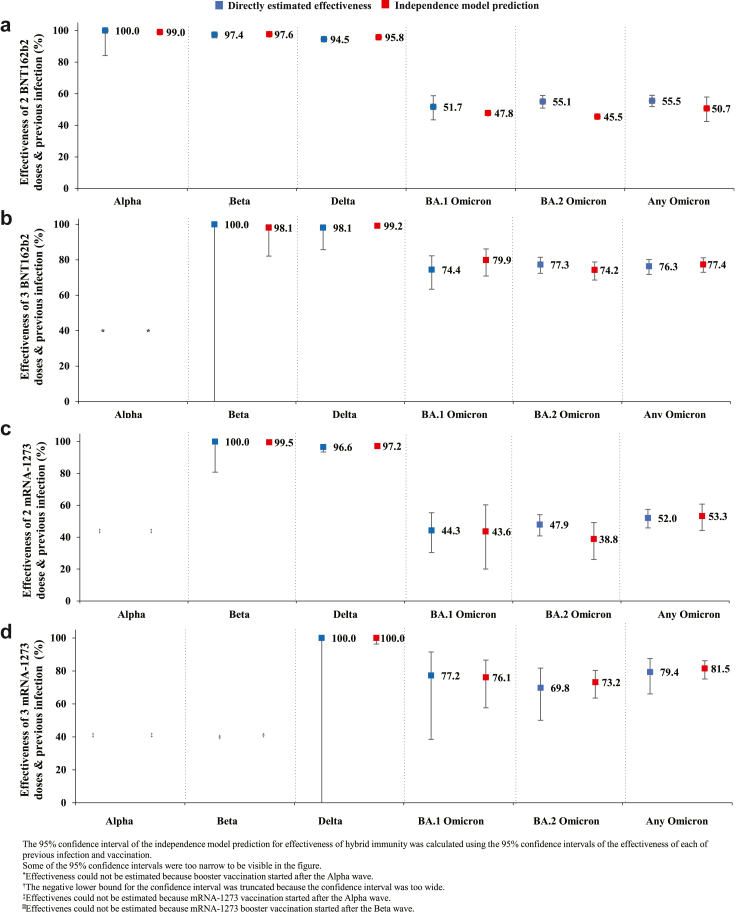
Estimated effectiveness of hybrid immunity against symptomatic Alpha, Beta, Delta, BA.1 Omicron, BA.2 Omicron, and any Omicron infection compared to predicted effectiveness against these infections assuming independence of the effects of previous infection immunity and vaccination immunity (independence model prediction).

Fig. 3 shows the directly estimated effectiveness measures of hybrid immunity in comparison to the predictions made by the independence model, that is assuming the effects of previous infection and vaccination act independently of each other. The estimates from both approaches were found to be very similar. The ICC was calculated to be 0.984 (95% CI: 0.962–0.994%). This ICC value suggests that the independence model accurately predicted the protection of hybrid immunity and with very high precision. Consequently, there is no evidence supporting either synergy or redundancy in the effects of natural infection immunity and vaccine immunity.

## Discussion

This study conducted a comprehensive assessment of the interplay between natural infection immunity and vaccine immunity in the pre-Omicron era, considering both the primary series and booster vaccinations. It specifically investigated how these immunological effects interact to generate the effect of hybrid immunity. The investigation encompassed an examination of hybrid immunity against the three primary variants of the pre-Omicron era, namely Alpha, Beta, and Delta, in the context of scarce evidence regarding the effects of immunity against the Beta variant, the most immune-evasive variant in the pre-Omicron era.^39^

Hybrid immunity provided higher protection against infection than each of natural infection or vaccination alone. This finding is consistent with epidemiological and laboratory studies indicating superior protection for hybrid immunity.^12^^,^^40^, ^41^, ^42^ Strikingly, a simple mathematical model accurately and precisely predicted the effectiveness of hybrid immunity from the individual effects of previous infection and vaccination by assuming that each acted independently of the other one. The combined effect of these two forms of protection reflected neither synergy nor redundancy of their individual biological effects.

These consistent findings, observed across various variants, both pre- and post-emergence of Omicron, and irrespective of vaccine type and dose number, suggests the existence of a generic immunological pattern applicable to immunity generated by the SARS-CoV-2 virus. This pattern may hold potential implications for optimizing protection against infection and severe COVID-19 through the development of diverse vaccine/booster formulations.

The study findings emphasize the significance of vaccination, even for individuals with a prior SARS-CoV-2 infection, as hybrid immunity consistently outperformed immunity derived solely from infection. Moreover, these findings highlight the importance of long-term monitoring of infected and vaccinated populations to gain a deeper understanding of how different forms of immunity, and subsequent exposures to these forms of immunity, interact and influence protection against infection and severe COVID-19. This is more relevant that the global population carries heterogeneous immune histories at present, and this heterogeneity in immune exposures will increase over time.

This study did not investigate the role of neutralizing antibodies as a potential explanation for these findings or being the correlate of protection,^43^ nor did it assess the long-term protection conferred by infection or vaccination. The generalizability of the study findings beyond the initial months following infection or vaccination remains uncertain,^15^^,^^44^ as does the specific contribution of neutralizing antibodies to the observed protection against infection. It is useful to explore the possibility that infection and vaccination independently induce the production of antibody titers, and that the combined effect of both titers may explain the observed enhanced protection associated with hybrid immunity. Further research is needed to investigate this hypothesis.

Protection of a pre-Omicron infection against reinfection with Alpha, Beta, or Delta was strong at ∼90%. This finding confirms the series of studies indicating strong protection for pre-Omicron infection against pre-Omicron reinfection, including on this same population.^1^, ^2^, ^3^, ^4^^,^^18^^,^^29^^,^^45^^,^^46^ Protection of primary-series vaccination against Alpha or Beta was also strong at >80% for both BNT162b2 and mRNA-1273, confirming earlier findings.^26^^,^^31^ Protection against Delta was lower, at ∼60% for BNT162b2 and at ∼70% for mRNA-1273, supporting earlier findings,^6^ but also reflecting the waning of primary-series vaccine protection by time of Delta dominance.^5^^,^^6^^,^^27^ Protection of the booster dose against Delta was strong at >90% for both vaccines, a consequence of the booster dose being recent, also supporting earlier findings.^13^

While there were differences in the protection of previous infection, vaccination, and hybrid immunity against infection, all of these forms of immunity had very strong protection against severe, critical, or fatal COVID-19, irrespective of variant, at >90%. This finding affirms the strong protection of any form of immunity against severe infection and that breakthrough infections, when they occur, are not likely to be severe. This finding is consistent with other findings indicating that reinfections are ∼90% less likely to be severe than primary infections,^45^^,^^47^ and that vaccination induces strong protection against severe COVID-19 that lasts longer than the vaccine’s protection against infection.^5^^,^^6^^,^^27^

This study has limitations. With the relatively young population of Qatar,^19^ our findings may not be generalizable to other countries where elderly citizens constitute a large proportion of the population. The study used an observational test-negative design, but bias can arise in such design in unexpected ways, or from unknown sources, such as subtle differences in test-seeking behavior or changes in the pattern of testing. Variant ascertainment was based on a time criteria of the variant that dominated incidence and not based on viral genome sequencing or genotyping of every infection. Few of the estimated effect sizes involved cells with zero counts and very wide 95% CIs, which raises concern about potential sparse-data bias.^48^

The hybrid effectiveness estimates were derived without considering the order of immunological events, specifically whether vaccination occurred before or after previous infection. This distinction could not be made due to small group sizes, as there were small number of cases of individuals being infected after vaccination during the pre-Omicron era. The investigation of factors such as the order of immunological events is being undertaken in other studies that encompass both the pre-Omicron and Omicron eras, with a focus on exploring immune imprinting effects.^15^^,^^49^^,^^50^

We examined the protective effects of previous infection compared to vaccination among groups that may differ in the time elapsed since their last immune conferring event. Both previous infection and vaccine-induced immunity wane over time,^5^^,^^45^^,^^51^^,^^52^ which could potentially affect the reliability of the comparison. However, this study was conducted in 2021, a year characterized by the rapid scale-up of vaccination and the presence of significant Alpha and Beta waves. The protection conferred by natural infection exhibited also a slow waning prior to the emergence of Omicron.^45^ Therefore, the time elapsed since the last immune conferring event may not have been a critical factor in this specific study, reducing the potential impact on the reliability of the comparison.

As an observational study, it is impossible to completely eliminate the possibility of unmeasured or uncontrolled confounding factors. While matching was done for several factors, it was not possible for other factors such as geography or occupation, as such data were unavailable. However, Qatar is essentially a city state and infection incidence was distributed across neighborhoods. Nationality, age, and sex provide a powerful proxy for socio-economic status in this country,^19^, ^20^, ^21^, ^22^ and thus matching by these factors may have also (partially) controlled for other factors such as occupation. This matching prescription had already been investigated in previous studies of different epidemiologic designs, and using control groups to test for null effects.^5^^,^^9^^,^^25^, ^26^, ^27^ These studies have supported that this prescription provides adequate control of the differences in infection exposure.^5^^,^^9^^,^^25^, ^26^, ^27^ Of note that case-control matching does not guarantee the elimination of confounding and may, in fact, introduce selection bias.^23^ Therefore, the low SMD in our study does not necessarily indicate the absence of confounding.^23^ The study was implemented on Qatar’s total population at a time of mass-scale PCR testing,^5^ perhaps minimizing the likelihood of bias.

Notwithstanding these limitations, findings are consistent with those of earlier studies that used different epidemiologic study designs on the same population.^1^^,^^3^, ^4^, ^5^, ^6^^,^^13^^,^^18^^,^^26^^,^^27^^,^^31^^,^^45^^,^^47^ Extensive sensitivity and additional analyses were conducted to investigate effects of potential bias in our earlier studies that used a similar methodology.^5^^,^^18^^,^^27^ These included different adjustments in the analysis and various study inclusion and exclusion criteria, to investigate whether effectiveness estimates could have been biased.^5^^,^^18^^,^^27^ These analyses supported the reliability of the approach of this study.^1^^,^^5^^,^^18^^,^^27^^,^^53^

In conclusion, all forms of natural and vaccine immunity prior to Omicron introduction provided strong protection against Alpha, Beta, and Delta infections, and very strong protection against severe COVID-19. Hybrid immunity of natural infection and vaccination provided higher protection against infection than that of natural infection or vaccination alone, regardless of variant. Remarkably, a simple mathematical model accurately and precisely predicted the effectiveness of hybrid immunity from the individual effects of previous infection and vaccination by assuming that each acted independently of the other one, with no synergy or redundancy. The study findings reinforce the importance of vaccination, even among individuals with a previous SARS-CoV-2 infection. However, considering the declining severity and fatality rates of infection,^54^ it is important to carefully evaluate and assess the risk-to-benefit ratio of vaccination for different population groups^55^ taking into account factors such as age and the presence of pre-existing conditions.^15^

## Contributors

HNA co-designed the study, performed the statistical analyses, and co-wrote the first draft of the article. HC co-designed the study and co-led the statistical analyses. LJA conceived and co-designed the study, co-led the statistical analyses, and co-wrote the first draft of the article. HNA, HC, and LJA accessed and verified all the data. PT and MRH designed and conducted multiplex, RT-qPCR variant screening and viral genome sequencing. PVC designed mass PCR testing to allow routine capture of SGTF variants and conducted viral genome sequencing. HY, HAK, and MS conducted viral genome sequencing. All authors (HNA, HC, HHA, PT, MRH, HMA, HAA-K, AAA-T, PVC, ZA-K, E-AK, AJ, AHK, ANL, RMS, HFA-R, GKN, MGA-K, AAB, HEA-R, MHA-T, AAK, RB, and LJA) contributed to data collection and acquisition, database development, discussion and interpretation of the results, and to the writing of the article. All authors have read and approved the final manuscript.

## Data sharing statement

The dataset of this study is a property of the Qatar Ministry of Public Health that was provided to the researchers through a restricted-access agreement that prevents sharing the dataset with a third party or publicly. The data are available under restricted access for preservation of confidentiality of patient data. Access can be obtained through a direct application for data access to Her Excellency the Minister of Public Health (https://www.moph.gov.qa/english/OurServices/eservices/Pages/Governmental-HealthCommunication-Center.aspx). The raw data are protected and are not available due to data privacy laws. Aggregate data are available within the paper and its supplementary information.

## Declaration of interests

Dr. Butt has received institutional grant funding from Gilead Sciences unrelated to the work presented in this paper. Otherwise, we declare no competing interests.
