# Supplementary material for: Effects of previous infection, vaccination, and hybrid immunity against symptomatic Alpha, Beta, and Delta SARS-CoV-2 infections: an observational study
Source: eBioMedicine. 2023 Jul 27;95:104734. doi: 10.1016/j.ebiom.2023.104734 (PMC10404859; doi:10.1016/j.ebiom.2023.104734)
Supplement: Multimedia component 1 [file mmc1.docx]

**Supplementary Appendix**

**Table of Contents**

[***Text S1*. Further details on methods.** 2](#_Toc138163198)

[*Data sources and testing* 2](#_Toc138163199)

[*Comorbidity classification* 4](#_Toc138163200)

[*Calculation of previous infection and vaccination effectiveness* 4](#_Toc138163201)

[***Text S2*. Laboratory methods and variant ascertainment.** 6](#_Toc138163202)

[*Real-time reverse-transcription polymerase chain reaction testing* 6](#_Toc138163203)

[*Classification of infections by variant type* 6](#_Toc138163204)

[***Text S3*. COVID-19 severity, criticality, and fatality classification.** 7](#_Toc138163205)

[***Table* *S1*.** Strengthening the Reporting of Observational Studies in Epidemiology (STROBE) checklist for case-control studies. 9](#_Toc138163206)

[***Figure* *S1*.** Daily number of newly diagnosed SARS-CoV-2 infections between January 15, 2021 and December 18, 2021. 11](#_Toc138163207)

[***Figure S2*.** Flowchart describing the population selection process for investigating effectiveness of previous pre-Omicron infection, vaccination with BNT162b2, and hybrid immunity against symptomatic Alpha, Beta, or Delta infections. 12](#_Toc138163208)

[***Figure S3*.** Flowchart describing the population selection process for investigating effectiveness of previous pre-Omicron infection, vaccination with mRNA-1273, and hybrid immunity against symptomatic Alpha, Beta, or Delta infections. 13](#_Toc138163209)

[***Table S2*.** Characteristics of matched cases and controls in samples used to estimate effectiveness against symptomatic Alpha, Beta, or Delta infections in the mRNA-1273 analysis. 14](#_Toc138163210)

[***Table S3.*** Effectiveness of previous pre-Omicron infection, vaccination with mRNA-1273, and hybrid immunity of previous infection and vaccination against symptomatic Alpha, Beta, or Delta infections and against severe, critical, or fatal COVID-19 due to infection with these variants. 16](#_Toc138163211)

[**References** 17](#_Toc138163212)

# ***Text S1*. Further details on methods.**

## *Data sources and testing*

Qatar’s national and universal public healthcare system uses the Cerner-system advanced digital health platform to track all electronic health record encounters of each individual in the country, including all citizens and residents registered in the national and universal public healthcare system. Registration in the public healthcare system is mandatory for citizens and residents.

The databases analyzed in this study are data-extract downloads from the Cerner-system that have been implemented on a regular (twice weekly) schedule since the onset of pandemic by the Business Intelligence Unit at Hamad Medical Corporation. Hamad Medical Corporation is the national public healthcare provider in Qatar. At every download all tests, coronavirus disease 2019 (COVID-19) vaccinations, hospitalizations related to COVID-19, and all death records regardless of cause are provided to the authors through .csv files. These databases have been analyzed throughout the pandemic not only for study-related purposes, but also to provide policymakers with summary data and analytics to inform the national response.

Every health encounter in the Cerner-system is linked to a unique individual through the HMC Number that links all records for this individual at the national level. Databases were merged and analyzed using the HMC Number to link all records whether for testing, vaccinations, hospitalizations, and deaths. All deaths in Qatar are tracked by the public healthcare system. All COVID-19-related healthcare was provided only in the public healthcare system. No private entity was permitted to provide COVID-19-related healthcare. COVID-19 vaccination was also provided only through the public healthcare system. These health records were tracked throughout the COVID-19 pandemic using the Cerner system. This system has been implemented in 2013, before the onset of the pandemic. Therefore, we had the health records related to this study for the full national cohort of citizens and residents throughout the pandemic.

Demographic details for every HMC Number (individual) such as sex, age, and nationality are collected upon issuing of the universal health card, based on the Qatar Identity Card, which is a mandatory requirement by the Ministry of Interior to every citizen and resident in the country.

Severe acute respiratory syndrome coronavirus 2 (SARS-CoV-2) testing in Qatar is done at a mass scale where close to 5% of the population are tested every week.(1, 2) All SARS-CoV-2 testing in any facility in this country is tracked nationally in one database, the national testing database. This database covers all testing in all locations and facilities throughout the country, whether public or private. Every polymerase chain reaction (PCR) test, regardless of location or setting, is classified on the basis of symptoms and the reason for testing (clinical symptoms, contact tracing, surveys or random testing campaigns, individual requests, routine healthcare testing, pre-travel, at port of entry, or other). Based on the distribution of the reason for testing, most of the tests that have been conducted in Qatar were conducted for routine reasons, such as being travel-related. About 75% of those diagnosed are also diagnosed not because of appearance of symptoms, but because of routine testing.(1, 2)

Qatar has unusually young, diverse demographics, in that only 9% of its residents are ≥50 years of age, and 89% are expatriates from over 150 countries.(3, 4) Further descriptions of the study population and these national databases were reported previously.(1, 2, 5-7)

## *Comorbidity classification*

Comorbidities were ascertained and classified based on the ICD-10 codes for chronic conditions as recorded in the electronic health record encounters of each individual in the Cerner-system national database that includes all citizens and residents registered in the national and universal public healthcare system. The public healthcare system provides healthcare to the entire resident population of Qatar free of charge or at heavily subsidized costs, including prescription drugs. With the mass expansion of this sector in recent years, facilities have been built to cater to specific needs of subpopulations. For example, tens of facilities have been built, including clinics and hospitals, in localities with high density of craft and manual workers.(8)

All encounters for each individual were analyzed to determine the comorbidity classification for that individual, as part of a recent national analysis to assess healthcare needs and resource allocation. The Cerner-system national database includes encounters starting from 2013, after this system was launched in Qatar. As long as each individual had at least one encounter with a specific comorbidity diagnosis since 2013, this person was classified with this comorbidity.

Individuals who have comorbidities but never sought care in the public healthcare system, or seek care exclusively in private healthcare facilities, were classified as individuals with no comorbidity due to absence of recorded encounters for them.

## *Calculation of previous infection and vaccination effectiveness*

Effectiveness measures and associated 95% CIs were calculated as 1-odds ratio (OR) of previous infection and/or vaccination among cases versus controls if the OR<1,(9, 10) and as 1/OR-1 if the OR was ≥1.(7, 11) The latter was done to ensure symmetric scale for both negative and positive effectiveness, ranging from -100%-100%, leading to easier and meaningful interpretation of effectiveness, regardless of being positive or negative.

# ***Text S2*. Laboratory methods and variant ascertainment.**

## *Real-time reverse-transcription polymerase chain reaction testing*

Nasopharyngeal and/or oropharyngeal swabs were collected for polymerase chain reaction (PCR) testing and placed in Universal Transport Medium (UTM). Aliquots of UTM were: 1) extracted on KingFisher Flex (Thermo Fisher Scientific, USA), MGISP-960 (MGI, China), or ExiPrep 96 Lite (Bioneer, South Korea) followed by testing with real-time reverse-transcription PCR (RT-qPCR) using TaqPath COVID-19 Combo Kits (Thermo Fisher Scientific, USA) on an ABI 7500 FAST (Thermo Fisher Scientific, USA); 2) tested directly on the Cepheid GeneXpert system using the Xpert Xpress SARS-CoV-2 (Cepheid, USA); or 3) loaded directly into a Roche cobas 6800 system and assayed with the cobas SARS-CoV-2 Test (Roche, Switzerland). The first assay targets the viral S, N, and ORF1ab gene regions. The second targets the viral N and E-gene regions, and the third targets the ORF1ab and E-gene regions.

All PCR testing was conducted at the Hamad Medical Corporation Central Laboratory or Sidra Medicine Laboratory, following standardized protocols.

## *Classification of infections by variant type*

Surveillance for the severe acute respiratory syndrome coronavirus 2 (SARS-CoV-2) variants in Qatar is based on viral genome sequencing and multiplex real-time reverse-transcription polymerase chain reaction (RT-qPCR) variant screening(12) of weekly collected random positive clinical samples,(2, 13-17) complemented by deep sequencing of wastewater samples.(15, 18, 19) Further details on the viral genome sequencing and multiplex RT-qPCR variant screening throughout the SARS-CoV-2 waves in Qatar can be found in previous publications.(1, 2, 5, 13-17, 20-25)

# ***Text S3*. COVID-19 severity, criticality, and fatality classification.**

Classification of Coronavirus Disease 2019 (COVID-19) case severity (acute-care hospitalizations),(26) criticality (intensive-care-unit hospitalizations),(26) and fatality(27) followed World Health Organization (WHO) guidelines. Assessments were made by trained medical personnel independent of study investigators and using individual chart reviews, as part of a national protocol applied to every hospitalized COVID-19 patient. Each hospitalized COVID-19 patient underwent an infection severity assessment every three days until discharge or death. We classified individuals who progressed to severe, critical, or fatal COVID-19 between the time of the documented infection and the end of the study based on their worst outcome, starting with death,(27) followed by critical disease,(26) and then severe disease.(26)

Severe COVID-19 disease was defined per WHO classification as a SARS-CoV-2 infected person with “oxygen saturation of <90% on room air, and/or respiratory rate of >30 breaths/minute in adults and children >5 years old (or ≥60 breaths/minute in children <2 months old or ≥50 breaths/minute in children 2-11 months old or ≥40 breaths/minute in children 1–5 years old), and/or signs of severe respiratory distress (accessory muscle use and inability to complete full sentences, and, in children, very severe chest wall indrawing, grunting, central cyanosis, or presence of any other general danger signs)”.(26) Detailed WHO criteria for classifying Severe acute respiratory syndrome coronavirus 2 (SARS-CoV-2) infection severity can be found in the WHO technical report.(26)

Critical COVID-19 disease was defined per WHO classification as a SARS-CoV-2 infected person with “acute respiratory distress syndrome, sepsis, septic shock, or other conditions that would normally require the provision of life sustaining therapies such as mechanical ventilation (invasive or non-invasive) or vasopressor therapy”.(26) Detailed WHO criteria for classifying SARS-CoV-2 infection criticality can be found in the WHO technical report.(26)

COVID-19 death was defined per WHO classification as “a death resulting from a clinically compatible illness, in a probable or confirmed COVID-19 case, unless there is a clear alternative cause of death that cannot be related to COVID-19 disease (e.g. trauma). There should be no period of complete recovery from COVID-19 between illness and death. A death due to COVID-19 may not be attributed to another disease (e.g. cancer) and should be counted independently of preexisting conditions that are suspected of triggering a severe course of COVID-19”. Detailed WHO criteria for classifying COVID-19 death can be found in the WHO technical report.(27)

# ***Table* *S1*.** Strengthening the Reporting of Observational Studies in Epidemiology (STROBE) checklist for case-control studies.

|  | Item No | Recommendation | Main text page |
| --- | --- | --- | --- |
| **Title and abstract** | 1 | (*a*) Indicate the study’s design with a commonly used term in the title or the abstract | Abstract |
|  |  | (*b*) Provide in the abstract an informative and balanced summary of what was done and what was found | Abstract |
| Introduction | | |  |
| Background/rationale | 2 | Explain the scientific background and rationale for the investigation being reported | Introduction |
| Objectives | 3 | State specific objectives, including any prespecified hypotheses | Introduction |
| Methods | | |  |
| Study design | 4 | Present key elements of study design | Methods (‘Study design’) |
| Setting | 5 | Describe the setting, locations, and relevant dates, including periods of recruitment, exposure, follow-up, and data collection | Methods (‘Study population and data sources’, ‘Study design’, & ‘Variant ascertainment’) & Texts S1, S2, & S3 in Appendix |
| Participants | 6 | (*a*) Give the eligibility criteria, and the sources and methods of case ascertainment and control selection. Give the rationale for the choice of cases and controls | Methods (‘Study population and data sources’ & ‘Study design’), Figures S2 & S3 in Appendix |
|  |  | (*b*) For matched studies, give matching criteria and the number of controls per case |  |
| Variables | 7 | Clearly define all outcomes, exposures, predictors, potential confounders, and effect modifiers. Give diagnostic criteria, if applicable | Methods (‘Study design’, ‘Variant ascertainment’, & ‘Statistical Analysis’) & Texts S1, S2, & S3 in Appendix |
| Data sources/ measurement | 8 | For each variable of interest, give sources of data and details of methods of assessment (measurement). Describe comparability of assessment methods if there is more than one group | Methods (‘Study population and data sources’, ‘Study design’, ‘Variant ascertainment’, & ‘Statistical analysis’), Table 1, & Texts S1-S3 & Table S2 in Appendix |
| Bias | 9 | Describe any efforts to address potential sources of bias | Methods (‘Study design’ & ‘Statistical analysis’) |
| Study size | 10 | Explain how the study size was arrived at | Figures S2 & S3 in Appendix |
| Quantitative variables | 11 | Explain how quantitative variables were handled in the analyses. If applicable, describe which groupings were chosen and why | Methods (‘Study design’ & ‘Statistical analysis’), Table 1, & Table S2 in Appendix |
| Statistical methods | 12 | (*a*) Describe all statistical methods, including those used to control for confounding | Methods (‘Statistical analysis’) |
|  |  | (*b*) Describe any methods used to examine subgroups and interactions | Methods (‘Statistical analysis’, paragraph 2) |
|  |  | (*c*) Explain how missing data were addressed | Not applicable, see Methods (‘Study population and data sources’) |
|  |  | (*d*) If applicable, explain how matching of cases and controls was addressed | Methods (‘Study design’, paragraph 2 & ‘Statistical analysis’, paragraph 2) |
|  |  | (*e*) Describe any sensitivity analyses | Not applicable |
| Results | | |  |
| Participants | 13 | (a) Report numbers of individuals at each stage of study—eg numbers potentially eligible, examined for eligibility, confirmed eligible, included in the study, completing follow-up, and analysed | Figures S2 & S3 in Appendix |
|  |  | (b) Give reasons for non-participation at each stage |  |
|  |  | (c) Consider use of a flow diagram |  |
| Descriptive data | 14 | (a) Give characteristics of study participants (eg demographic, clinical, social) and information on exposures and potential confounders | Results (‘Study population’), Table 1 & Table S2 in Appendix |
|  |  | (b) Indicate number of participants with missing data for each variable of interest | Not applicable, see Methods (‘Study population and data sources’) |
| Outcome data | 15 | Report numbers in each exposure category, or summary measures of exposure | Results ‘(‘Effectiveness against symptomatic Alpha infection’, ‘Effectiveness against symptomatic Beta infection’, ‘Effectiveness against symptomatic Delta infection’, ‘Effectiveness against severe, critical, or fatal COVID-19’, & ‘Hybrid immunity protection: Directly estimated versus independence-model prediction’), Figures 1, 2, 3, Table 2, & Table S3 in Appendix |
| Main results | 16 | (*a*) Give unadjusted estimates and, if applicable, confounder-adjusted estimates and their precision (eg, 95% confidence interval). Make clear which confounders were adjusted for and why they were included | Results ‘(‘Effectiveness against symptomatic Alpha infection’, ‘Effectiveness against symptomatic Beta infection’, ‘Effectiveness against symptomatic Delta infection’, ‘Effectiveness against severe, critical, or fatal COVID-19’, & ‘Hybrid immunity protection: Directly estimated versus independence-model prediction’), Figures 1, 2, 3, Table 2, & Table S3 in Appendix |
|  |  | (*b*) Report category boundaries when continuous variables were categorized | Table 1 & Table S2 in Appendix |
|  |  | (*c*) If relevant, consider translating estimates of relative risk into absolute risk for a meaningful time period | Not applicable |
| Other analyses | 17 | Report other analyses done—eg analyses of subgroups and interactions, and sensitivity analyses | Not applicable |
| Discussion | | |  |
| Key results | 18 | Summarise key results with reference to study objectives | Discussion, paragraphs 1-4 & paragraphs 6-7 |
| Limitations | 19 | Discuss limitations of the study, taking into account sources of potential bias or imprecision. Discuss both direction and magnitude of any potential bias | Discussion, paragraphs 5 & paragraphs 8-12 |
| Interpretation | 20 | Give a cautious overall interpretation of results considering objectives, limitations, multiplicity of analyses, results from similar studies, and other relevant evidence | Discussion, paragraph 13 |
| Generalisability | 21 | Discuss the generalisability (external validity) of the study results | Discussion, paragraph 5, 8 & 11 |
| Other information | | |  |
| Funding | 22 | Give the source of funding and the role of the funders for the present study and, if applicable, for the original study on which the present article is based | Funding |

# ***Figure* *S1*.** Daily number of newly diagnosed SARS-CoV-2 infections between January 15, 2021 and December 18, 2021.

**
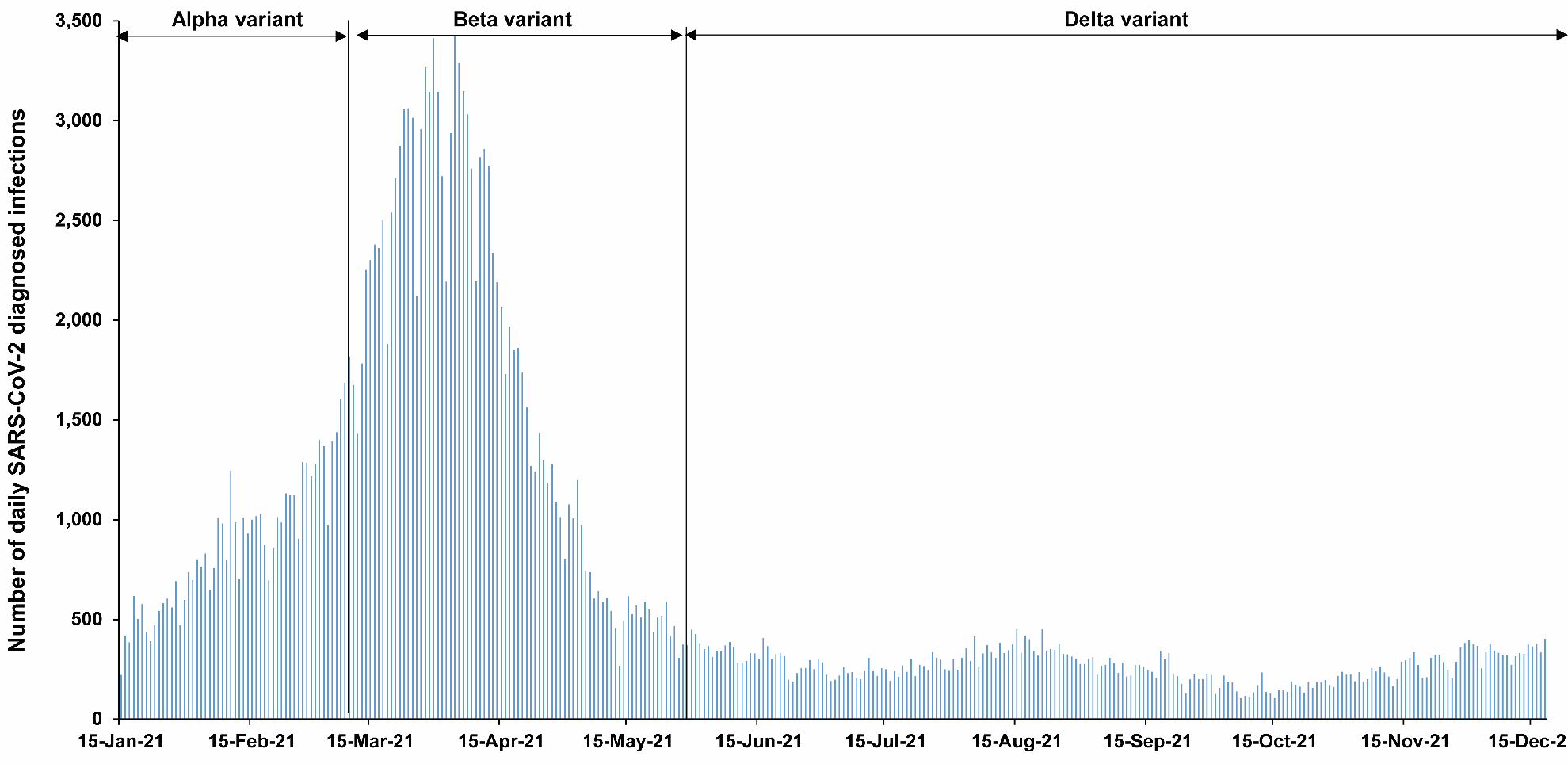
**

# ***Figure S2*.** Flowchart describing the population selection process for investigating effectiveness of previous pre-Omicron infection, vaccination with BNT162b2, and hybrid immunity against symptomatic Alpha, Beta, or Delta infections.


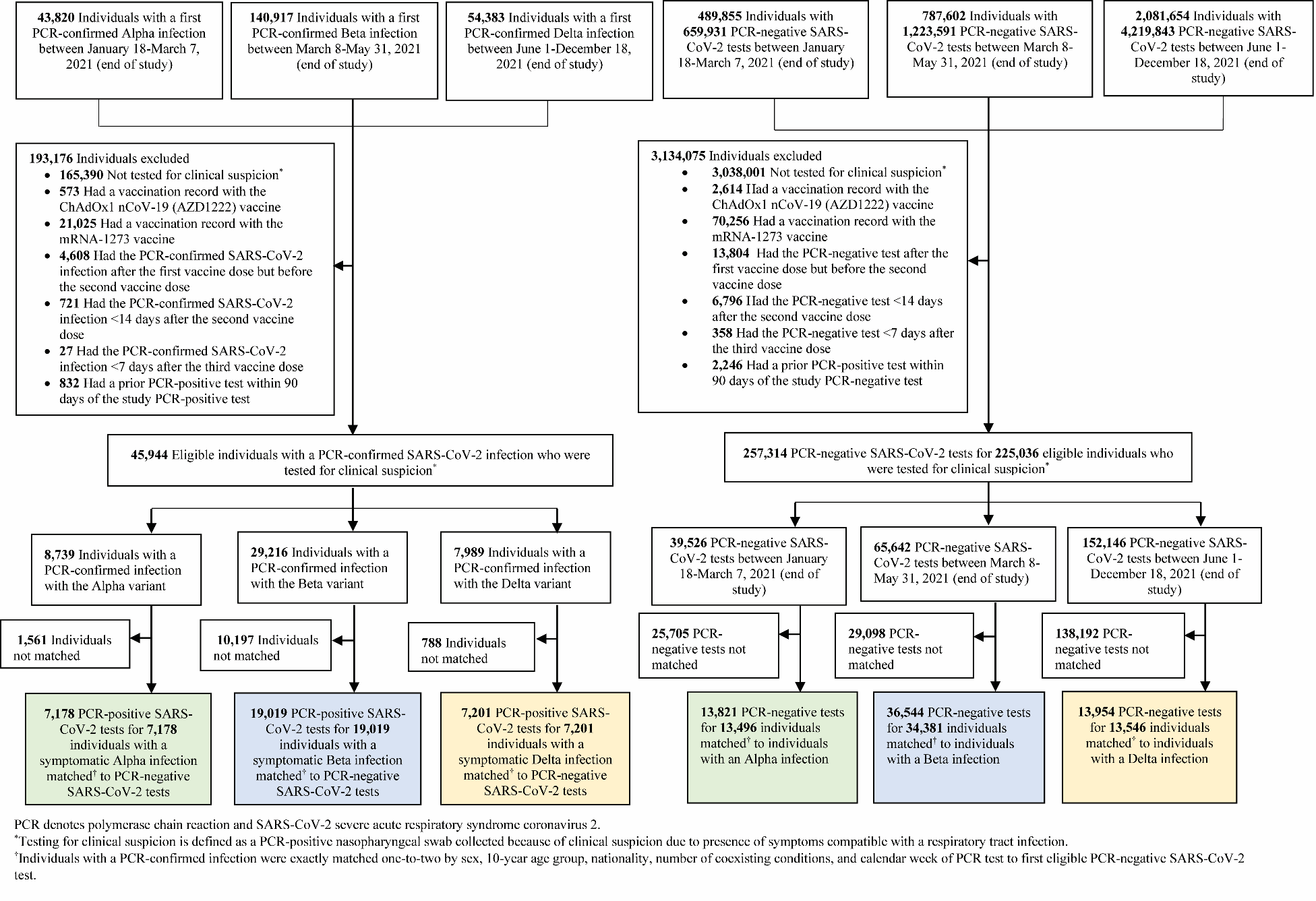


# ***Figure S3*.** Flowchart describing the population selection process for investigating effectiveness of previous pre-Omicron infection, vaccination with mRNA-1273, and hybrid immunity against symptomatic Alpha, Beta, or Delta infections.


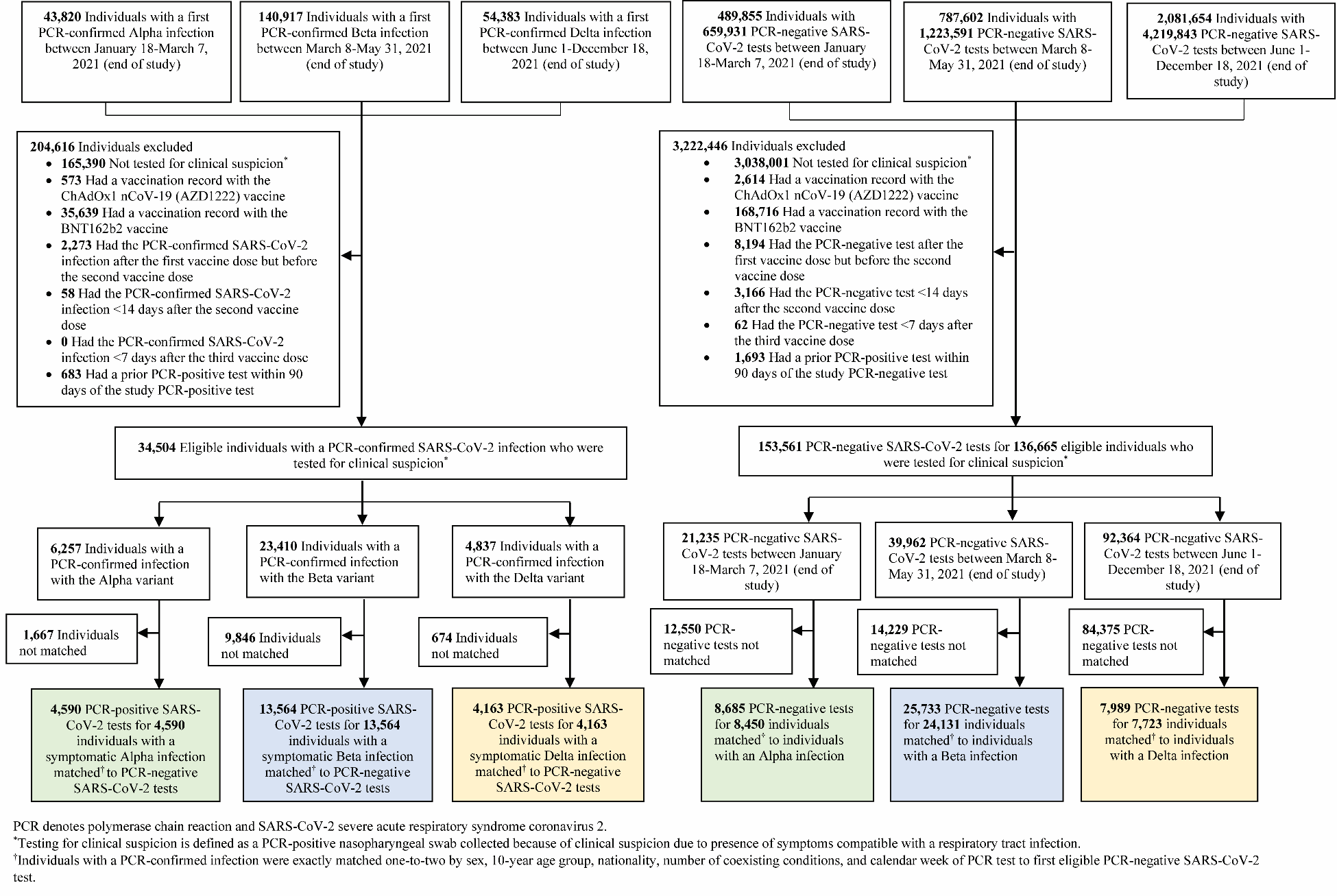


# ***Table S2*.** Characteristics of matched cases and controls in samples used to estimate effectiveness against symptomatic Alpha, Beta, or Delta infections in the mRNA-1273 analysis.

| Characteristics | Effectiveness against symptomatic Alpha infection | | | Effectiveness against symptomatic Beta infection | | | Effectiveness against any symptomatic Delta infection | | |
| --- | --- | --- | --- | --- | --- | --- | --- | --- | --- |
|  | **Cases^*^**  **(PCR-positive)** | **Controls^*^**  **(PCR-negative)** | SMD^†^ | **Cases^*^**  **(PCR-positive)** | **Controls^*^**  **(PCR-negative)** | SMD^†^ | **Cases^*^**  **(PCR-positive)** | **Controls^*^**  **(PCR-negative)** | SMD^†^ |
|  | **N=4,590** | **N=8,685** |  | **N=13,564** | **N=25,733** |  | **N=4,163** | **N=7,989** |  |
| Median age (IQR) — years | 32 (24-40) | 32 (24-39) | 0.04^‡^ | 30 (22-37) | 31 (22-38) | 0.03^‡^ | 16 (7-33) | 16 (6-33) | 0.03^‡^ |
| Age group — no. (%) |  |  |  |  |  |  |  |  |  |
| <10 years | 670 (14.6) | 1,295 (14.9) | 0.04 | 2,376 (17.5) | 4,564 (17.7) | 0.04 | 1,495 (35.9) | 2,927 (36.6) | 0.03 |
| 10-19 years | 220 (4.8) | 409 (4.7) |  | 722 (5.3) | 1,338 (5.2) |  | 689 (16.6) | 1,321 (16.5) |  |
| 20-29 years | 909 (19.8) | 1,740 (20.0) |  | 3,109 (22.9) | 5,990 (23.3) |  | 594 (14.3) | 1,151 (14.4) |  |
| 30-39 years | 1,613 (35.1) | 3,120 (35.9) |  | 4,520 (33.3) | 8,735 (33.9) |  | 816 (19.6) | 1,562 (19.6) |  |
| 40-49 years | 873 (19.0) | 1,610 (18.5) |  | 2,041 (15.0) | 3,784 (14.7) |  | 399 (9.6) | 743 (9.3) |  |
| 50-59 years | 242 (5.3) | 413 (4.8) |  | 633 (4.7) | 1071 (4.2) |  | 121 (2.9) | 203 (2.5) |  |
| 60-69 years | 48 (1.0) | 75 (0.9) |  | 110 (0.8) | 161 (0.6) |  | 35 (0.8) | 58 (0.7) |  |
| 70+ years | 15 (0.3) | 23 (0.3) |  | 53 (0.4) | 90 (0.3) |  | 14 (0.3) | 24 (0.3) |  |
| Sex |  |  |  |  |  |  |  |  |  |
| Male | 2,845 (62.0) | 5,400 (62.2) | 0.04 | 8,766 (64.6) | 16,790 (65.2) | 0.01 | 2,186 (52.5) | 4,220 (52.8) | 0.01 |
| Female | 1,745 (38.0) | 3,285 (37.8) |  | 4,798 (35.4) | 8,943 (34.8) |  | 1,977 (47.5) | 3,769 (47.2) |  |
| Nationality^§^ |  |  |  |  |  |  |  |  |  |
| Bangladeshi | 321 (7.0) | 622 (7.2) | 0.05 | 1,351 (10.0) | 2,619 (10.2) | 0.04 | 225 (5.4) | 439 (5.5) | 0.04 |
| Egyptian | 472 (10.3) | 894 (10.3) |  | 852 (6.3) | 1,591 (6.2) |  | 417 (10.0) | 798 (10.0) |  |
| Filipino | 492 (10.7) | 946 (10.9) |  | 1,335 (9.8) | 2,559 (9.9) |  | 187 (4.5) | 363 (4.5) |  |
| Indian | 1,265 (27.6) | 2,467 (28.4) |  | 2,771 (20.4) | 5,414 (21.0) |  | 413 (9.9) | 811 (10.2) |  |
| Nepalese | 234 (5.1) | 455 (5.2) |  | 1,003 (7.4) | 1,952 (7.6) |  | 91 (2.2) | 176 (2.2) |  |
| Pakistani | 273 (5.9) | 507 (5.8) |  | 612 (4.5) | 1,132 (4.4) |  | 122 (2.9) | 235 (2.9) |  |
| Qatari | 501 (10.9) | 989 (11.4) |  | 2,243 (16.5) | 4,408 (17.1) |  | 1,553 (37.3) | 3,069 (38.4) |  |
| Sri Lankan | 130 (2.8) | 244 (2.8) |  | 492 (3.6) | 919 (3.6) |  | 56 (1.3) | 109 (1.4) |  |
| Sudanese | 127 (2.8) | 223 (2.6) |  | 460 (3.4) | 850 (3.3) |  | 136 (3.3) | 253 (3.2) |  |
| Other nationalities^¶^ | 775 (16.9) | 1,338 (15.4) |  | 2,445 (18.0) | 4,289 (16.7) |  | 963 (23.1) | 1,736 (21.7) |  |
| Coexisting conditions |  |  |  |  |  |  |  |  |  |
| 0 | 3,518 (76.6) | 6,824 (78.6) | 0.05 | 10,874 (80.2) | 21,046 (81.8) | 0.04 | 3,066 (73.6) | 5,943 (74.4) | 0.03 |
| 1 | 656 (14.3) | 1,189 (13.7) |  | 1,783 (13.1) | 3,195 (12.4) |  | 801 (19.2) | 1,530 (19.2) |  |
| 2 | 221 (4.8) | 367 (4.2) |  | 504 (3.7) | 824 (3.2) |  | 168 (4.0) | 289 (3.6) |  |
| 3+ | 195 (4.2) | 305 (3.5) |  | 403 (3.0) | 668 (2.6) |  | 128 (3.1) | 227 (2.8) |  |
| PCR test calendar month^**^ |  |  |  |  |  |  |  |  |  |
| January | 820 (17.9) | 1,558 (17.9) | 0.00 | 0 (0.0) | 0 (0.0) | 0.01 | 0 (0.0) | 0 (0.0) | 0.02 |
| February | 2,781 (60.6) | 5,247 (60.4) |  | 0 (0.0) | 0 (0.0) |  | 0 (0.0) | 0 (0.0) |  |
| March | 989 (21.5) | 1,880 (21.6) |  | 5,862 (43.2) | 11,289 (43.9) |  | 0 (0.0) | 0 (0.0) |  |
| April | 0 (0.0) | 0 (0.0) |  | 6,019 (44.4) | 11,244 (43.7) |  | 0 (0.0) | 0 (0.0) |  |
| May | 0 (0.0) | 0 (0.0) |  | 1,683 (12.4) | 3,200 (12.4) |  | 0 (0.0) | 0 (0.0) |  |
| June | 0 (0.0) | 0 (0.0) |  | 0 (0.0) | 0 (0.0) |  | 620 (14.9) | 1,176 (14.7) |  |
| July | 0 (0.0) | 0 (0.0) |  | 0 (0.0) | 0 (0.0) |  | 482 (11.6) | 902 (11.3) |  |
| August | 0 (0.0) | 0 (0.0) |  | 0 (0.0) | 0 (0.0) |  | 709 (17.0) | 1,403 (17.6) |  |
| September | 0 (0.0) | 0 (0.0) |  | 0 (0.0) | 0 (0.0) |  | 450 (10.8) | 851 (10.7) |  |
| October | 0 (0.0) | 0 (0.0) |  | 0 (0.0) | 0 (0.0) |  | 367 (8.8) | 742 (9.3) |  |
| November | 0 (0.0) | 0 (0.0) |  | 0 (0.0) | 0 (0.0) |  | 908 (21.8) | 1,729 (21.6) |  |
| December | 0 (0.0) | 0 (0.0) |  | 0 (0.0) | 0 (0.0) |  | 627 (15.1) | 1,186 (14.8) |  |

IQR denotes interquartile range, PCR polymerase chain reaction, and SMD standardized mean difference.

^*^Cases and controls were matched exactly one-to-two by sex, 10-year age group, nationality, number of coexisting conditions, and calendar week of PCR test.

^†^SMD is the difference in the mean of a covariate between groups divided by the pooled standard deviation. An SMD of ≤0.1 indicates adequate matching.

^‡^SMD is for the mean difference between groups divided by the pooled standard deviation.

^§^Nationalities were chosen to represent the most populous groups in Qatar.

^¶^These comprise 41 other nationalities in Qatar among cases and controls in the analysis for effectiveness against symptomatic Alpha infection, 58 other nationalities among cases and controls in the analysis for effectiveness against symptomatic Beta infection, and 34 other nationalities among cases and controls in the analysis for effectiveness against symptomatic Delta infection.

^**^Cases and controls were exactly matched using calendar week of PCR test, but we opted to report the distribution by calendar month for brevity. Accordingly, some cases and controls who were tested in the same week may appear in different calendar months.

# ***Table S3.*** Effectiveness of previous pre-Omicron infection, vaccination with mRNA-1273, and hybrid immunity of previous infection and vaccination against symptomatic Alpha, Beta, or Delta infections and against severe, critical, or fatal COVID-19 due to infection with these variants.

| **Analyses** | **Cases**  **(PCR-positive)^*^** | | **Controls**  **(PCR-negative)^*^** | | **Effectiveness against symptomatic infection (95% CI)** | **Cases**  **(Severe, critical, or fatal COVID-19)**^†^ | | **Controls**  **(PCR- negative)**^†^ | | **Effectiveness against severe, critical, or fatal COVID-19 (95% CI)** |
| --- | --- | --- | --- | --- | --- | --- | --- | --- | --- | --- |
|  | **Exposed** | **Unexposed**^‡^ | **Exposed** | **Unexposed**^‡^ |  | **Exposed** | **Unexposed**^‡^ | **Exposed** | **Unexposed**^‡^ |  |
| **Alpha symptomatic infection^§^** | | | | | | | | | | |
| Previous infection and no vaccination | 28 | 4,562 | 428 | 8,257 | 88.3 (82.8 to 92.1) | 0 | 293 | 63 | 1,010 | 100.0 (94.0 to 100.0)^¶^ |
| Two doses and no previous infection | 0 | 4,562 | 0 | 8,257 | - | 0 | 293 | 0 | 1,010 | - |
| Two doses and previous infection | 0 | 4,562 | 0 | 8,257 | - | 0 | 293 | 0 | 1,010 | - |
| Three doses and no previous infection | 0 | 4,562 | 0 | 8,257 | - | 0 | 293 | 0 | 1,010 | - |
| Three doses and previous infection | 0 | 4,562 | 0 | 8,257 | - | 0 | 293 | 0 | 1,010 | - |
| **Beta symptomatic infection^§^** | | | | | | | | | | |
| Previous infection and no vaccination | 97 | 13,459 | 1,340 | 24,096 | 87.5 (84.6 to 89.9) | 1 | 1,133 | 269 | 4,114 | 98.8 (91.4 to 99.8) |
| Two doses and no previous infection | 8 | 13,459 | 276 | 24,096 | 96.0 (91.6 to 98.1) | 0 | 1,133 | 49 | 4,114 | 100.0 (92.2 to 100.0)^¶^ |
| Two doses and previous infection | 0 | 13,459 | 21 | 24,096 | 100.0 (80.8 to 100.0)^¶^ | 0 | 1,133 | 2 | 4,114 | 100.0 (-81.2 to 100.0)^¶^ |
| Three doses and no previous infection | 0 | 13,459 | 0 | 24,096 | - | 0 | 1,133 | 0 | 4,114 | - |
| Three doses and previous infection | 0 | 13,459 | 0 | 24,096 | - | 0 | 1,133 | 0 | 4,114 | - |
| **Delta symptomatic infection^§^** | | | | | | | | | | |
| Previous infection and no vaccination | 44 | 3,631 | 613 | 5,586 | 90.4 (86.8 to 93.0) | 0 | 125 | 35 | 244 | 100.0 (88.9 to 100.0)^¶^ |
| Two doses and no previous infection | 477 | 3,631 | 1,521 | 5,586 | 71.0 (66.4 to 74.9) | 3 | 125 | 176 | 244 | 97.9 (91.2 to 99.5) |
| Two doses and previous infection | 11 | 3,631 | 249 | 5,586 | 96.6 (93.5 to 98.2) | 0 | 125 | 19 | 244 | 100.0 (78.6 to 100.0)^¶^ |
| Three doses and no previous infection | 0 | 3,631 | 15 | 5,586 | 100.0 (72.1 to 100.0)^¶^ | 0 | 125 | 1 | 244 | 100.0 (-97.4 to 100.0)^¶^ |
| Three doses and previous infection | 0 | 3,631 | 5 | 5,586 | 100.0 (-8.4 to 100.0)^¶^ | 0 | 125 | 1 | 244 | 100.0 (-97.4 to 100.0)^¶^ |

CI denotes confidence interval, COVID-19 coronavirus disease 2019, and PCR polymerase chain reaction.

**^*^**Cases and controls were exactly matched one-to-two by sex, 10-year age group, nationality, number of coexisting conditions, and calendar week of PCR test.

^†^Cases and controls were exactly matched one-to-five by sex, 10-year age group, nationality, number of coexisting conditions, and calendar week of PCR test.

^‡^Unexposed was defined as no previous infection and no vaccination.

**^§^**A symptomatic infection was defined as a PCR-positive nasopharyngeal swab that was obtained because of the presence of symptoms consistent with a respiratory tract infection. Effectiveness was estimated with the use of a test-negative, case-control study design. COVID-19 severity, criticality, and fatality were defined according to World Health Organization guidelines.

^¶^The 95% confidence interval was estimated with the use of McNemar’s test because of zero events among exposed cases. Since n:1 matching was employed, the number of pairs was considered as ‘n’. This approach provided only an approximate estimate for the 95% CI in these specific situations.

# **References**

1. Altarawneh HN, Chemaitelly H, Ayoub HH, Tang P, Hasan MR, Yassine HM, et al. Effects of Previous Infection and Vaccination on Symptomatic Omicron Infections. N Engl J Med. 2022;387(1):21-34.

2. Chemaitelly H, Tang P, Hasan MR, AlMukdad S, Yassine HM, Benslimane FM, et al. Waning of BNT162b2 Vaccine Protection against SARS-CoV-2 Infection in Qatar. N Engl J Med. 2021;385(24):e83.

3. Planning and Statistics Authority-State of Qatar. Qatar Monthly Statistics. Available from: <https://www.psa.gov.qa/en/pages/default.aspx>. Accessed on: May 26, 2020. 2020.

4. Abu-Raddad LJ, Chemaitelly H, Ayoub HH, Al Kanaani Z, Al Khal A, Al Kuwari E, et al. Characterizing the Qatar advanced-phase SARS-CoV-2 epidemic. Sci Rep. 2021;11(1):6233.

5. Abu-Raddad LJ, Chemaitelly H, Ayoub HH, AlMukdad S, Yassine HM, Al-Khatib HA, et al. Effect of mRNA Vaccine Boosters against SARS-CoV-2 Omicron Infection in Qatar. N Engl J Med. 2022;386(19):1804-16.

6. Chemaitelly H, Faust JS, Krumholz H, Ayoub H, Tang P, Coyle P, et al. Short- and longer-term all-cause mortality among SARS-CoV-2- infected persons and the pull-forward phenomenon in Qatar. medRxiv. 2023:2023.01.29.23285152.

7. Chemaitelly H, Ayoub HH, Tang P, Coyle P, Yassine HM, Al Thani AA, et al. Long-term COVID-19 booster effectiveness by infection history and clinical vulnerability and immune imprinting: a retrospective population-based cohort study. Lancet Infect Dis. 2023.

8. Al-Thani MH, Farag E, Bertollini R, Al Romaihi HE, Abdeen S, Abdelkarim A, et al. SARS-CoV-2 Infection Is at Herd Immunity in the Majority Segment of the Population of Qatar. Open Forum Infect Dis. 2021;8(8):ofab221.

9. Jackson ML, Nelson JC. The test-negative design for estimating influenza vaccine effectiveness. Vaccine. 2013;31(17):2165-8.

10. Ayoub HH, Tomy M, Chemaitelly H, Altarawneh HN, Coyle P, Tang P, et al. Estimating protection afforded by prior infection in preventing reinfection: Applying the test-negative study design. medRxiv. 2022:2022.01.02.22268622.

11. Tseng HF, Ackerson BK, Bruxvoort KJ, Sy LS, Tubert JE, Lee GS, et al. Effectiveness of mRNA-1273 vaccination against SARS-CoV-2 omicron subvariants BA.1, BA.2, BA.2.12.1, BA.4, and BA.5. Nature Communications. 2023;14(1):189.

12. Vogels C, Fauver J, Grubaugh N. Multiplexed RT-qPCR to screen for SARS-COV-2 B.1.1.7, B.1.351, and P.1 variants of concern V.3. dx.doi.org/10.17504/protocols.io.br9vm966. 2021(June 6, 2021).

13. Abu-Raddad LJ, Chemaitelly H, Butt AA, National Study Group for Covid Vaccination. Effectiveness of the BNT162b2 Covid-19 Vaccine against the B.1.1.7 and B.1.351 Variants. N Engl J Med. 2021;385(2):187-9.

14. Chemaitelly H, Yassine HM, Benslimane FM, Al Khatib HA, Tang P, Hasan MR, et al. mRNA-1273 COVID-19 vaccine effectiveness against the B.1.1.7 and B.1.351 variants and severe COVID-19 disease in Qatar. Nat Med. 2021;27(9):1614-21.

15. National Project of Surveillance for Variants of Concern and Viral Genome Sequencing. Qatar viral genome sequencing data. Data on randomly collected samples. <https://www.gisaid.org/phylodynamics/global/nextstrain/> 2021 [Available from: <https://www.gisaid.org/phylodynamics/global/nextstrain/>.

16. Benslimane FM, Al Khatib HA, Al-Jamal O, Albatesh D, Boughattas S, Ahmed AA, et al. One Year of SARS-CoV-2: Genomic Characterization of COVID-19 Outbreak in Qatar. Front Cell Infect Microbiol. 2021;11:768883.

17. Hasan MR, Kalikiri MKR, Mirza F, Sundararaju S, Sharma A, Xaba T, et al. Real-Time SARS-CoV-2 Genotyping by High-Throughput Multiplex PCR Reveals the Epidemiology of the Variants of Concern in Qatar. Int J Infect Dis. 2021;112:52-4.

18. Saththasivam J, El-Malah SS, Gomez TA, Jabbar KA, Remanan R, Krishnankutty AK, et al. COVID-19 (SARS-CoV-2) outbreak monitoring using wastewater-based epidemiology in Qatar. Sci Total Environ. 2021;774:145608.

19. El-Malah SS, Saththasivam J, Jabbar KA, K KA, Gomez TA, Ahmed AA, et al. Application of human RNase P normalization for the realistic estimation of SARS-CoV-2 viral load in wastewater: A perspective from Qatar wastewater surveillance. Environ Technol Innov. 2022;27:102775.

20. Tang P, Hasan MR, Chemaitelly H, Yassine HM, Benslimane FM, Al Khatib HA, et al. BNT162b2 and mRNA-1273 COVID-19 vaccine effectiveness against the SARS-CoV-2 Delta variant in Qatar. Nat Med. 2021;27(12):2136-43.

21. Altarawneh HN, Chemaitelly H, Hasan MR, Ayoub HH, Qassim S, AlMukdad S, et al. Protection against the Omicron Variant from Previous SARS-CoV-2 Infection. N Engl J Med. 2022;386(13):1288-90.

22. Chemaitelly H, Ayoub HH, AlMukdad S, Coyle P, Tang P, Yassine HM, et al. Duration of mRNA vaccine protection against SARS-CoV-2 Omicron BA.1 and BA.2 subvariants in Qatar. Nat Commun. 2022;13(1):3082.

23. Qassim SH, Chemaitelly H, Ayoub HH, AlMukdad S, Tang P, Hasan MR, et al. Effects of BA.1/BA.2 subvariant, vaccination and prior infection on infectiousness of SARS-CoV-2 omicron infections. J Travel Med. 2022;29(6).

24. Altarawneh HN, Chemaitelly H, Ayoub HH, Hasan MR, Coyle P, Yassine HM, et al. Protective Effect of Previous SARS-CoV-2 Infection against Omicron BA.4 and BA.5 Subvariants. N Engl J Med. 2022;387(17):1620-2.

25. Chemaitelly H, Tang P, Coyle P, Yassine HM, Al-Khatib HA, Smatti MK, et al. Protection against Reinfection with the Omicron BA.2.75 Subvariant. N Engl J Med. 2023;388(7):665-7.

26. World Health Organization (WHO). Living guidance for clinical management of COVID-19. Aavailable from: <https://www.who.int/publications/i/item/WHO-2019-nCoV-clinical-2021-2>. Accessed on: February 27, 2023. 2021.

27. World Health Organization (WHO). International Guidelines for Certification and Classification (Coding) of COVID-19 as Cause of Death. Available from: <https://www.who.int/publications/m/item/international-guidelines-for-certification-and-classification-(coding)-of-covid-19-as-cause-of-death>. Accessed on: February 27, 2023. 2020.
